# The Use of Whole Genome and Exome Sequencing for Newborn Screening: Challenges and Opportunities for Population Health

**DOI:** 10.3389/fped.2021.663752

**Published:** 2021-07-19

**Authors:** Audrey C. Woerner, Renata C. Gallagher, Jerry Vockley, Aashish N. Adhikari

**Affiliations:** ^1^Department of Pediatrics, University of Pittsburgh Medical Center Children's Hospital of Pittsburgh, University of Pittsburgh School of Medicine, Pittsburgh, PA, United States; ^2^Department of Pediatrics, University of California, San Francisco, San Francisco, CA, United States; ^3^Department of Human Genetics, University of Pittsburgh Graduate School of Public Health, Pittsburgh, PA, United States; ^4^Institute for Human Genetics, University of California, San Francisco, San Francisco, CA, United States; ^5^Artificial Intelligence Lab, Illumina Inc, Foster City, CA, United States

**Keywords:** newborn screening, whole genome sequencing, whole exome sequencing, next-generation sequencing, recommended uniform screening panel

## Abstract

Newborn screening (NBS) is a population-based program with a goal of reducing the burden of disease for conditions with significant clinical impact on neonates. Screening tests were originally developed and implemented one at a time, but newer methods have allowed the use of multiplex technologies to expand additions more rapidly to standard panels. Recent improvements in next-generation sequencing are also evolving rapidly from first focusing on individual genes, then panels, and finally all genes as encompassed by whole exome and genome sequencing. The intersection of these two technologies brings the revolutionary possibility of identifying all genetic disorders in newborns, allowing implementation of therapies at the optimum time regardless of symptoms. This article reviews the history of newborn screening and early studies examining the use of whole genome and exome sequencing as a screening tool. Lessons learned from these studies are discussed, along with technical, ethical, and societal challenges to broad implementation.

## Introduction

Medical screening is an important part of health care and public health initiatives. The purpose of a medical screening test is to identify a medical condition early, ideally in the pre-symptomatic phase, so that appropriate treatment can be initiated to decrease morbidity and mortality. Screening is particularly indicated for medical conditions in which early treatment is more effective than treatment in later stages of the condition. Population screening adds a requirement of broader societal benefit in addition to benefit for the individual. Newborn screening (NBS) was cited by the United States Centers for Disease Control and Prevention as one of the most impactful public health initiatives of the twentieth century and in the twenty first century has undergone significant expansion through improved techniques of high-throughput biochemical analysis of targeted analytes, enzymatic activities, and specific molecular defects. It now stands on the verge of incorporating increasingly large-scale molecular sequencing to detect a growing number of disorders. This article will provide a history of NBS, specifically focusing on the elements pertinent to implementation of whole exome and whole genome sequencing (WES and WGS, respectively), review efforts to date on the use of sequencing for NBS, and discuss the challenges for the future for larger incorporation in NBS programs.

## History of Newborn Screening

### Inborn Errors of Metabolism

In 1935, phenylpyruvic acid was reported in the urine of a subset of individuals with intellectual disability and later termed phenylketonuria (PKU) (1). Subsequently, the possibility of treatment for PKU with restriction of dietary phenylalanine was postulated, and in 1954, the first report was published of a child with PKU treated with a diet restricted in phenylalanine who showed significant clinical improvement and elimination of phenylpyruvate in the urine (2). While the detection of urine phenylpyruvate could be used to diagnose PKU, it was not until Robert Guthrie developed a bacterial inhibition assay in 1958 that early, rapid, and accurate testing of phenylalanine levels for diagnosis and management of PKU became a reality (3). The final piece fell into place in 1961 when a filter paper method for collecting samples as a dried blood spot (DBS) was developed that is used in NBS to this day (4). Screening for PKU began in New York in 1961, and in 1963, Massachusetts became the first state to mandate NBS for PKU (3, 5). The introduction of NBS for PKU was clearly a turning point for the disease, as it allowed normal development in identified babies who otherwise would have suffered devastating neurodevelopmental symptoms. Over time, it was determined that NBS for PKU not only identifies classic PKU due to phenylalanine hydroxylase deficiency but also non-PKU hyperphenylalaninemia and tetrahydrobiopterin deficiency, all of which are characterized by elevation of a single metabolite, phenylalanine.

The success of NBS for PKU allowing early identification and intervention that prevented intellectual disability led to the hope that expansion of NBS would translate to similar improvement for other appropriate conditions (6, 7).

### The Wilson and Jungner Principles

Given a growing interest in expanding NBS to more disorders, a meeting sponsored by the World Health Organization (WHO) in 1968 led to the publication of the Wilson and Jungner guidelines outlining 10 principles to guide development and implementation of a screening test and provided the framework for the further development of NBS programs (Table 1) (8). Necessary characteristics of a screening test include the availability of an economical method of identification of important health problems for which treatment is available, as well as the infrastructure to provide such treatment. These guidelines clearly went beyond the principle that a disease should be added to NBS based on technical ability and formed the basis of NBS expansion for several decades.

**Table 1:** Wilson and Jungner principles and 2006 ACMG criteria.

**Wilson and Jungner 10 principles of a screening test (8)**
1. The condition sought should be an important health problem.
2. There should be an accepted treatment for patients with recognized disease.
3. Facilities for diagnosis and treatment should be available.
4. There should be a recognizable latent or early symptomatic stage.
5. There should be a suitable test or examination.
6. The test should be acceptable to the population
7. The natural history of the condition, including development from latent to declared disease, should be adequately understood.
8. There should be an agreed policy on whom to treat as patients.
9. The cost of case-finding (including diagnosis and treatment of patients diagnosed) should be economically balanced in relation to possible expenditure on medical care as a whole.
10. Case-finding should be a continuing process and not a “once and for all” project.
**2006 ACMG criteria (9)**
1. The clinical characteristics of the condition
a. Incidence of the condition b. Signs and symptoms were clinically apparent in the first 48 h of life c. Burden of disease if untreated d. Individual benefit of early intervention e. Family and societal benefits of early intervention f. Prevention of mortality through early diagnosis and treatment
2. The analytical characteristics of the test
a. Sensitive and specific screening test algorithm b. Test performed on DBS or a simple point-of-care method c. High throughput (more than 200 per day) d. Cost <$1 per test per condition e. Multiple analytes relevant to a single condition detected in the same run f. Other conditions detected by the same analytes g. Multiple conditions can be detected by the same test
3. Diagnosis, follow-up, treatment, and management of the condition a. Cost and availability of treatment b. Potential of existing treatment to prevent negative consequences of the condition c. Availability of diagnostic confirmation and acute management d. Simplicity of therapy

### Expansion of NBS Beyond Inborn Errors of Metabolism

While inborn errors of metabolism (IEM) were the initial focus of NBS, the scope of NBS quickly began to expand. In 1973, a method for identifying hemoglobinopathies from DBS was developed, and the first NBS for hemoglobinopathies began in New York in 1975 (10). Yet another milestone in NBS was achieved in 1987 when a process for extracting DNA from DBS was developed, and its use as a second-tier test to confirm the diagnosis of sickle cell disease after an initial positive NBS with isoelectric focusing (IEF) or high-performance liquid chromatography (HPLC) was reported (11, 12). Second-tier DNA testing is necessary to identify sickle cell disease, as multiple hemoglobinopathies are identified with IEF and HPLC (10).

Newborn screening for congenital hypothyroidism was first introduced in 1974 and represents another triumph in NBS akin to that of PKU, allowing early identification, treatment, and prevention of intellectual disability using a new technology, immunodetection (13). Shortly thereafter, NBS for a second endocrinopathy, congenital adrenal hyperplasia (CAH), was introduced using an immunoassay to identify the most common type of CAH, 21-hydroxylase deficiency (14). Newborn screening for CAH prevented neonatal death in the salt-wasting forms through early diagnosis and appropriate treatment, though CAH screening via immunoassay alone had a high false positive rate, particularly in premature infants (14).

Newborn screening for cystic fibrosis (CF) was introduced in 1988 and, similar to the two-tiered approach for hemoglobinopathy screening, utilized DNA extraction from DBS and assessment for common pathogenic variants in the *CFTR* gene after a positive first-tier screen, in this case with immunoreactive trypsinogen (IRT) as the first step in screening (15).

## New Technologies and Expanded Newborn Screening Targets

### Tandem Mass Spectrometry

**Figure 1:**
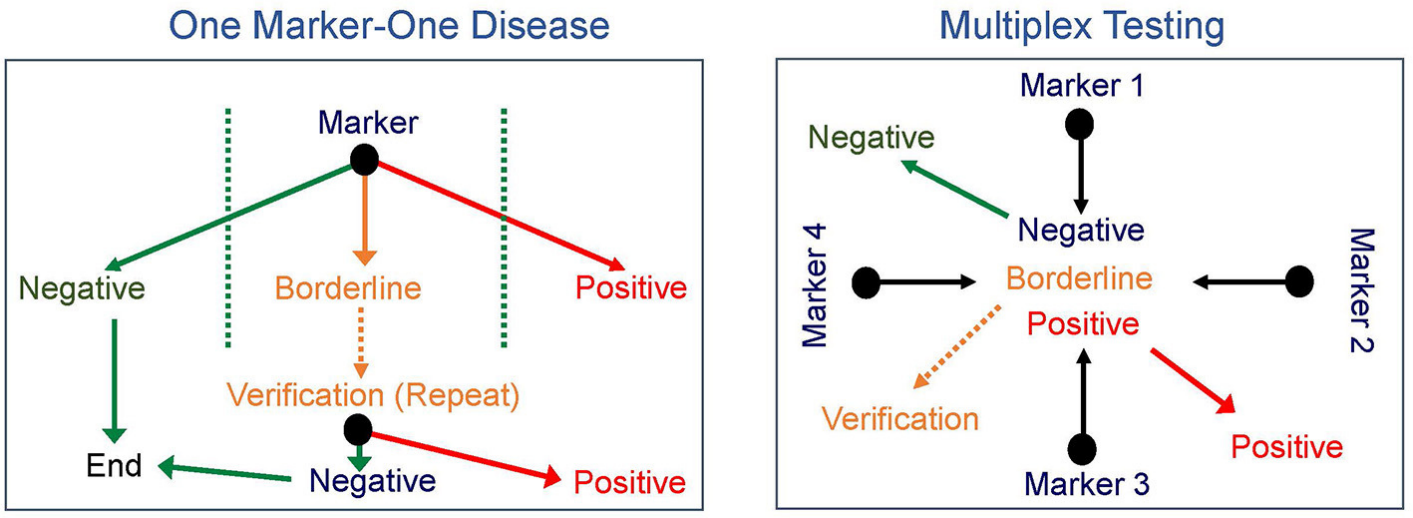
Newborn screening paradigms. Newborn screening progressed from one marker for each disease (left) to multiplex testing with multiple markers identified from a single test (right) broadening the scope of initial screening tests and significantly increasing the differential diagnosis of a positive newborn screen.

The early successes of NBS were all based on identifying one disease at a time, though as noted for PKU and as is true for other disorders, such as galactosemia, sometimes more than one disease can be diagnosed. This paradigm required the development and implementation of a new test for every proposed disorder added to screening. The next major advance in NBS came in the 1990's with the introduction of tandem mass spectrometry (MS/MS), which allows rapid simultaneous screening for multiple IEM using a single test (16–18). The concept of multiple markers for multiple disorders not only broadened the scope of initial screening tests but also significantly increased the differential diagnosis of a positive newborn screen (Figure 1). However, with this advancement in technology also came additional challenges. The technology was beyond that in use by most public NBS programs. Moreover, while MS/MS identified IEM that conformed to Wilson and Jungner principles for NBS disorders, it also identified others that do not meet these criteria (8, 16). For example, medium-chain acyl-CoA dehydrogenase (MCAD) deficiency is a disorder of fatty acid oxidation first identified in the late 1970s in a subset of children presenting with Reye syndrome-like features and abnormal urinary metabolites (19, 20). Prior to newborn screening for MCAD deficiency, affected individuals presented with acute crises characterized by hypoglycemia, lethargy, vomiting, seizures, hepatomegaly, and liver dysfunction, progressing to coma and death with a reported mortality rate of 20–30% in individuals diagnosed on a clinical basis (21, 22). In those who survived, an additional 40% showed evidence of significant neurological damage as a sequela of hypoglycemic crises (21, 23). The specific enzymatic defect was identified in 1982, and NBS for MCAD deficiency became a reality in 1990 when MS/MS on DBS became available (19, 20). The treatment for MCAD deficiency is avoidance of prolonged fasting and administration of IV dextrose if oral intake cannot be maintained due to intercurrent illness. After NBS for MCAD deficiency was introduced, morbidity and mortality decreased significantly, with a recent study reporting a mortality rate of 3.5% (20, 24). Clearly, the advent of NBS by MS/MS has saved lives and has a substantial beneficial effect on the natural history of MCAD deficiency. However, MS/MS also identifies short-chain acyl-CoA dehydrogenase deficiency (SCAD), another defect of fatty acid oxidation, which is now considered an asymptomatic biochemical condition and does not meet the Wilson and Jungner criteria (25). Other examples of disorders with significant clinical impact such as isovaleric acidemia were also balanced by disorders ultimately shown to have a low risk of clinical symptoms (3-methylcrotonyl-CoA carboxylase deficiency). Furthermore, other identified diseases were less amendable to early intervention (for example, mitochondrial trifunctional protein deficiency), though screening led to earlier diagnosis and avoidance of an extended diagnostic odyssey, providing an important benefit to families and allowing for genetic counseling for the couple and other family members.

### Tiered Testing Strategies

Tiered testing strategies, including utilization of MS/MS, have led to improved positive predictive value of abnormal screens (9, 26). For example, steroid profiling via MS/MS as a second-tier test after a positive NBS for CAH has been shown to decrease the false positive rate of the initial test (15). Screening for congenital hypothyroidism with thyroxine (T4) is highly sensitive but not particularly specific; the addition of thyrotropin (TSH) as a second-tier test in those neonates with low T4 improves the specificity and decreases the false positive rate (9, 27). Other examples of tiered testing strategies include DNA testing on DBS after positive screens for CF, hemoglobinopathies, and MCAD deficiency (9, 10, 15). It is important to distinguish tiered screening testing strategies from diagnostic testing. A positive NBS, with or without second-tier testing, requires follow-up diagnostic testing. In some cases, the technology may be the same used in the initial screening test (e.g., MS/MS for fatty acid oxidation disorders); however, confirmatory diagnostic testing is still indicated as the cut-off values for screening tests are different than those of diagnostic testing (9). In addition, confirmatory diagnostic testing often utilizes additional methodologies not included in the initial NBS test (e.g., urine organic acid analysis, enzyme analysis, and/or DNA sequencing).

## The Recommended Uniform Screening Panel

As more states began utilizing MS/MS for NBS, it became possible to screen for a larger number of disorders, and the variability in NBS among states increased (9). Recognizing the increasing complexity of the NBS landscape, in the late 1990's, the US Health Resource and Services Administration (HRSA) requested that the American Academy of Pediatrics (AAP) review the current state of affairs of NBS in the United States and provide recommendations for improvement. The AAP Newborn Screening Task Force concluded that there was a need for consistency and equity among NBS programs in the United States (28). In response, HRSA charged the American College of Medical Genetics and Genomics (ACMG) with developing a national framework for NBS and, specifically, with the development of a uniform panel of conditions as well as model policies, procedures, minimum standards, methods for expansion of NBS programs, as well as quality and oversight on a national level (9).

The ACMG developed a scoring system with which to evaluate each condition to be included on a recommended uniform screening panel, based on the following categories (9):

1. The clinical characteristics of the condition;
2. The analytical characteristics of the test; and
3. Diagnosis, follow-up, treatment, and management of the condition.

Each category included specific criteria upon which the tests were scored, outlined in Table 1. Based on the sum of scores from each category, a condition was then assigned to one of three groups, namely, the core panel, the secondary targets (differential diagnosis of core panel disorders), and conditions not appropriate for NBS (9). The ACMG also recommended standardization of reporting language, reporting standards, as well as improved oversight, long-term data collection, quality improvement, follow-up, and funding (9). With the inclusion of secondary targets, the ACMG broadened the original Wilson and Jungner criteria definition of benefit to include not only direct benefit for the individual being screened in the form of a specific treatment but also benefit to the individual screened, the family, and society derived from identification and management of disorders for which a specific treatment may not exist (9, 29, 30).

In 2008, the Advisory Committee on Heritable Disorders in Newborns and Children (ACHDNC), established by the Department of Health and Human Services (DHHS) in 2003, was given the responsibility to complete regular evidence-based systematic review of and recommendations for conditions to be included on the Recommended Universal Newborn Screening Panel (RUSP), which is based on the 2006 ACMG expert report outlining principles and processes for uniform NBS as well as the original Wilson and Jungner criteria (8, 9, 29, 31). Newborn screening in all states is mandatory, with limited ability for parents to opt out of testing (32). The current core and secondary targets can be found on the ACHDNC website at Recommended Uniform Screening Panel | Official web site of the U.S. Health Resources & Services Administration (hrsa.gov) (33).

### Point-of-Care NBS

Additional methods for NBS fall outside the DBS paradigm for testing. The first point-of-care NBS began with neonatal early hearing detection and intervention screening in Hawaii in 1990 and is now part of the RUSP (34). Identification of critical congenital heart disease by pulse oximetry screening, another point-of-care NBS test, was added in 2011 (35).

### Primary DNA NBS

Newborn screening for severe combined immunodeficiency (SCID) began in the United States in 2008, was added to the RUSP in 2010, and ushered in a new era of primary DNA screening (36). DNA extracted from DBS had been used for second-tier testing for sickle cell disease (SCD) utilizing PCR and allele-specific oligonucleotide hybridization, as SCD is due to a single specific beta-globin gene point mutation (12). NBS for SCID utilizes DNA extracted from DBS as the primary screen; quantitative PCR is used to assess DNA copy number (10, 15, 36). Severe combined immunodeficiency is characterized by decreased amounts of T- and/or B-cells and leads to the inability of the body to effectively fight infections (36). Without appropriate treatment, such as hematopoietic stem cell transplant or enzyme replacement, death due to overwhelming infections inevitably occurs. Severe combined immunodeficiency can be effectively screened for with the identification of decreased T-cell receptor excision circles (TRECs), which are small pieces of leftover DNA formed through DNA recombination in the process of T-cell maturation (36). Low numbers of TRECs identified on NBS indicate inadequate T-cells and trigger secondary testing with flow cytometry to identify the specific deficiency, as NBS for SCID also identifies other non-SCID T-cell deficiencies (36).

A more recent example of NBS utilizing DNA extracted from DBS as a primary target is *SMN1*-related spinal muscular atrophy (SMA), which was added to the RUSP in 2018 (31, 37). *SMN1*-related SMA is a progressive disorder characterized by muscle weakness due to loss of anterior horn cells and has a broad phenotypic spectrum ranging from a severe neonatal presentation with weakness, hypotonia, respiratory failure, and death in early infancy to an adult-onset presentation of muscle weakness without respiratory insufficiency and with a normal lifespan (31). Treatment for *SMN1*-related SMA has recently become available and slows or prevents the progression of the disorder once therapy is initiated, thus making it an ideal candidate for NBS (31). Screening identifies a common gene deletion, and as for SCID, the primary screen is a quantitative PCR technique; combining SCID and SMA in one newborn screen assay has been proposed (37). For both of these conditions, DNA is used for the primary screen but neither employs DNA sequencing. However, as a result of the introduction of SCID and SMA newborn screening, NBS programs now routinely extract DNA from DBS, allowing the introduction of other DNA-based assays, such as DNA sequencing. This and improvements that allow high-throughput DNA sequencing with a rapid turnaround time have led to an increased interest in the use of DNA sequencing as a primary NBS test.

## Whole Exome and Whole Genome Sequencing in Disease Diagnosis

The production of the map of the human genome promised a new era in medicine, one in which genetic and genomic information would be used routinely in health care (38). With an available map, advanced DNA sequencing techniques that are high throughput and low cost offered the promise of the personalized genome, that is, sequencing of an individual's genome, in order to provide a personal health benefit (39). In the two decades since the completion of the human genome, the utility of DNA sequencing for health benefit has been demonstrated multiple times and is now routine in some areas of medicine. The first use of individual genome information for disease diagnosis occurred in 2009 at the University of Wisconsin for a child with severe inflammatory bowel disease. Sequencing of his entire genome and DNA variant analysis yielded an answer, immunodeficiency due to a pathogenic variant in the *XIAP* gene (40). This test costs $75,000 and required 4 months of analysis (41). It resulted in life-saving treatment; based on this genetic diagnosis, the child was successfully treated for his debilitating disease with a cord blood transplant. Additional confirmation of the power of next-generation sequencing (NGS) technology came from a proof-of-principle paper in which exome sequencing identified the correct genetic diagnosis for four individuals with the rare disorder Freeman–Sheldon syndrome (42). Shortly thereafter, next-generation sequencing and DNA variant analysis of four individuals with Miller syndrome, which lacked a causative disease gene at that time, resulted in the identification of that gene, *DHODH* (43). Exome sequencing remains a method for disease diagnosis and for disease gene discovery (44, 45).

## Whole Exome and Whole Genome Sequencing in Disease Screening

### Newborn Sequencing in Genomic Medicine and Public Health

The power of exome sequencing and computational analysis to identify new disease genes and diagnose individuals with rare disorders suggested other uses for this technology including pre-conception carrier testing and NBS (46). The ability to extract DNA from dried blood spots (11) and the development of high-throughput technologies for DNA sequencing have led to the use of next-generation sequencing in NBS in follow-up testing after an abnormal enzyme or analyte primary screen and have held out the promise of the use of this as a primary screen, especially for those early-onset treatable disorders that lack a current NBS test (11, 47–52). Recognizing the need for research in the area of DNA sequencing and newborn health, the NIH funded the Newborn Sequencing in Genomic Medicine and Public Health (NSIGHT) network in the early 2010's (53). The three key research questions to be addressed were as follows: (1) For disorders currently screened in newborns, how can genomic sequencing replicate or augment known newborn screening results? Can sequencing replace current modalities? (2) What knowledge could genomic sequencing provide about conditions not currently screened for in newborns? (3) What additional clinical information could be learned from genomic sequencing relevant to the clinical care of newborns (53)? The four funded projects addressed these questions through separate study designs and patient populations (53). Two projects included cases with abnormal newborn screen results. In the North Carolina Newborn Exome Sequencing for Universal Screening (NC NEXUS) project, healthy newborns (61 subjects) and infants and children <5 years of age with known abnormal newborn screening results (17 subjects) or with hearing loss (28 subjects) were enrolled for exome sequencing. Analysis was blinded to phenotype, and analyzed genes included those with childhood-onset medically actionable disorders (NBS-NGS, 466 genes, all subjects) and those with diagnostic findings (affected subjects, additional indication-based genes analyzed). This project included randomization to an arm in which parents could decide whether to learn additional information from the genomic analysis including low or no actionability childhood-onset conditions, high actionability adult-onset conditions, and carrier status for recessive disorders (54). The NBSeq project performed exome sequencing retrospectively on DNA obtained from dried blood spots of cases with known IEM diagnosed through conventional newborn screening in California. These were de-identified samples and included cases with false positive newborn screens. The role of exome sequencing as a primary or secondary test for IEM was assessed (55). The two additional NSIGHT projects addressed the utility of WGS in sick newborns and the role of WES in sick and healthy newborns (BabySeq) and did not have a primary newborn screening aim (53).

The NC NEXUS project found that NBS-NGS was 88% sensitive for cases with an abnormal newborn screen for an IEM and 18% sensitive for the hearing loss cohort (54). Four individuals had abnormal NBS-NGS results not identified by other methods, including female heterozygote status for OTC deficiency in a child with PKU and heterozygous status for a known pathogenic variant in *LDLR* causing autosomal dominant familial hypercholesterolemia in a child with a known family history of this. Two children in the hearing loss cohort had additional clinically relevant findings, one with a variant in *DSC2* causing autosomal dominant arrhythmogenic right ventricular dysplasia and one with two variants in the gene associated with factor XI deficiency (54). These findings addressed the second aim of the NSIGHT consortium.

The NBSeq project found that DNA could not substitute as a primary newborn screen for disorders currently screened for by analyte, due to insufficient sensitivity (88%) and specificity (94%), but that DNA testing could be beneficial in follow-up testing in reducing follow-up of false positive results and in identifying disease diagnosis, which address the first aim of the NSIGHT consortium and demonstrate that DNA sequencing cannot substitute for current conventional screening but can augment it (55).

In the BabySeq project, which did not have a primary NBS component, three cases were found through WES to have newborn screening-related disorders missed on conventional screening. They were partial biotinidase deficiency (not associated with symptoms in most cases), non-classical congenital adrenal hyperplasia, and post-lingual *KCNQ4* hearing loss (56). These findings addressed the third aim of the NSIGHT consortium, by demonstrating that non-classical forms of some disorders can be identified, though there is no current information regarding sensitivity for these.

The NSIGHT projects have demonstrated the current capabilities and the gaps to be addressed to improve the performance of NGS in NBS. Importantly, despite technological advances, the Ethics and Policy Board of NSIGHT recommended against genomic sequencing of all babies at birth and called for a nuanced use of genomic technologies, taking into account the contexts of screening vs. diagnosis and the contexts of clinical care, public health, and direct to consumer testing (57).

### Practical Aspects of Genomic Testing for NBS

A crucial issue in genomic medicine is the large number of DNA variants in each individual. Depending on the context, narrowing the number of genes to be evaluated can be critical for improving test interpretation. Therefore, targeted testing has been proposed in carrier screening and NBS. This can also affect coverage of the individual genes assessed. In 2015, Naylor and colleagues reported the performance of two targeted next-generation sequencing (TNGS) platforms in two clinical contexts. They used targeted DNA sequencing either through WES with an *in silico* gene filter for 126 genes or through a next-generation sequencing panel, NBDx, which included a DNA capture step for exons of the 126-NBDx gene panel. These TNGS platforms were used both after an abnormal newborn screen and for ill infants in the neonatal intensive care unit (NICU). The authors compared these two platforms in the retrospective evaluation of 36 individuals with known IEM from the Amish and Mennonite communities. While the NBDx gene panel had advantages over WES, an important result from this study in a homogeneous population is that only 27 of 36 disorders were correctly identified in the experimental cohort in the absence of clinical information (50). This paper not only demonstrated the technical feasibility of obtaining DNA for NGS from dried blood spots and of developing a rapid and cost-effective test applicable to newborns with abnormal NBS and ill infants in the NICU, but also highlighted the limitation of DNA sequence information alone for disease diagnosis and the importance of clinical information to aid DNA variant interpretation. The authors suggest the possible use of NGS as a primary NBS in the future.

In 2017, the utility of NBS by WGS, rather than WES, was shown taking advantage of two DNA sequencing studies in newborns in the Inova Health System in Virginia (58). This study used two different WGS platforms on which 163 genes were analyzed. In contrast to the WES retrospective proof-of-principle study in a small number of affected individuals from the Amish and Mennonite communities, this study was conducted in an ancestrally diverse population of almost 1,700 newborns. Importantly, and unrealistically for a NBS public health program, parents were also sequenced, allowing phasing of variants for autosomal recessive disorders. Variants were classified using the ACMG criteria of pathogenic, likely pathogenic, benign, likely benign, and variant of uncertain significance (59). Only pathogenic and likely pathogenic variants were called. Importantly, exon coverage differed by gene and sequencing technology. While WGS did identify two cases missed on Virginia state NBS (hemoglobin SC disease and Duarte variant galactosemia, the latter a non-disease), the conclusion of this paper was that conventional NBS could not be replaced by WGS as NBS identified 4/5 affected neonates in the cohort and WGS identified only 2/5. This team suggested periodically reassessing NBS and WGS and using a larger cohort in later studies. The benefits of WGS in NBS were lower false positives than conventional NBS, resolution of inconclusive NBS results, distinguishing the correct disorder in cases of ambiguity with NBS results, and decreased numbers of follow-up samples required for preterm infants.

Next-generation DNA sequencing has been assessed as a primary newborn screen in Korea, where the use of a 307-gene panel for 159 disorders, including 60 neonatal IEM, was assessed. The study also addressed turnaround time (TAT), cost, and variant interpretation. They sequenced 103 subjects, 81 affected individuals and 22 controls. Remarkably, for the affected individuals, only 12% of causal variants were annotated in databases. Crucially, for the 307 genes, each subject had 8.6 variants, for 3.4 diseases; thus, manual curation and clinical information were required for each subject. Eighty-eight percent of the variants were non-disease causing (60). Variant interpretation was identified as a critical limiting factor in the use of TNGS as a primary screen in this work.

The UK is investing significantly in genomic sequencing, and the role of NGS in NBS in their National Health Service has been reported (52). They demonstrated that screening for five genes (*ACADM, PAH, TSHR, CFTR*, and *HBB*) in the National Health Service in the UK is feasible. The genes chosen for the study corresponded to disorders already on the NBS panel, specifically MCAD deficiency, PKU, congenital hypothyroidism, and sickle cell disease, respectively. They achieved a TAT of 4 days and could process 1,000 samples per week (a typical NBS lab processes 50,000 samples per year) but suggested that cost might be prohibitive as the cost of the current NBS test is 25 pounds, and the NGS test would cost 62 pounds or more. A consideration of adding diseases based on merit and not technologies was also recommended.

These papers and those of the NSIGHT network, discussed here and outlined in Table 2, demonstrate that while technically feasible, perhaps even with sufficient TAT to be appropriate for NBS, the high cost and limitations of variant detection (intronic variants, regulatory regions, copy number variants, structural variants, and trinucleotide repeats), variant interpretation and of annotated disease databases remain a significant barrier to the use of DNA as a primary screen. The contrast between the success of DNA in diagnosis and the difficulties in the use of DNA in screening highlights that the lack of a phenotype to guide variant interpretation remains the chief limitation of WES and WGS in NBS. In the next sections, we will examine technical aspects of NGS interpretation, especially as it relates to NBS.

**Table 2:** Summary of studies on next-generation sequencing for newborn screening.

**References**	**Study type**	**Platform**	**Number of genes assessed**	**Samples**	**Study population**	**Sensitivity**	**Specificity**
Bhattacharjee et al. (50)	Retrospective	NGS gene panel – NBDx; and WES	126	36 subjects with known IEM – proband only	Amish and Mennonite	75% without clinical information; 94% with clinical information	Not addressed
Bodian et al. (58)	Retrospective	WGS – Illumina or complete genomics	163	1,696 neonates – trios	Family trios enrolled at Inova Fairfax Hospital	88.6% concordance of NBS and WGS	Not addressed – for recessive disorders 2.9% with uncertain WGS results compared to 0.013% for NBS
Cho et al. (60)	Retrospective	WES	307 total, 65 related to NBS	103 patients 81 known patients 10 carriers 12 negative controls	Patients at Yonsei Severance Hospital, Republic of Korea	92.5% – with clinical information	Not addressed
van Campen et al. (52)	Proof of principle to address feasibility including cost and TAT	NGS gene panel – NBS2	5	Healthy adults	Adults in the UK	Analytic sensitivity 100%, disease samples not assessed	Analytic specificity 99.96%, disease samples not assessed
Roman et al. (54)	Prospective for the healthy cohort; retrospective for the affected cohorts	WES	466	106 newborns	61 healthy 17 with an IEM 28 with hearing loss	88% for IEM	Not addressed
Adhikari et al. (55)	Retrospective	WES	78	1,012 individuals in the test set: 674 affected with an IEM and 338 unaffected and false positive on MS/MS NBS	IEM-affected individuals from a birth cohort of 4.5 million newborns over 8.5 years in California	88–93.7% after clinical review of cases	98.4%

## Current Technologies to Detect Genetic Variants

**Figure 2:**
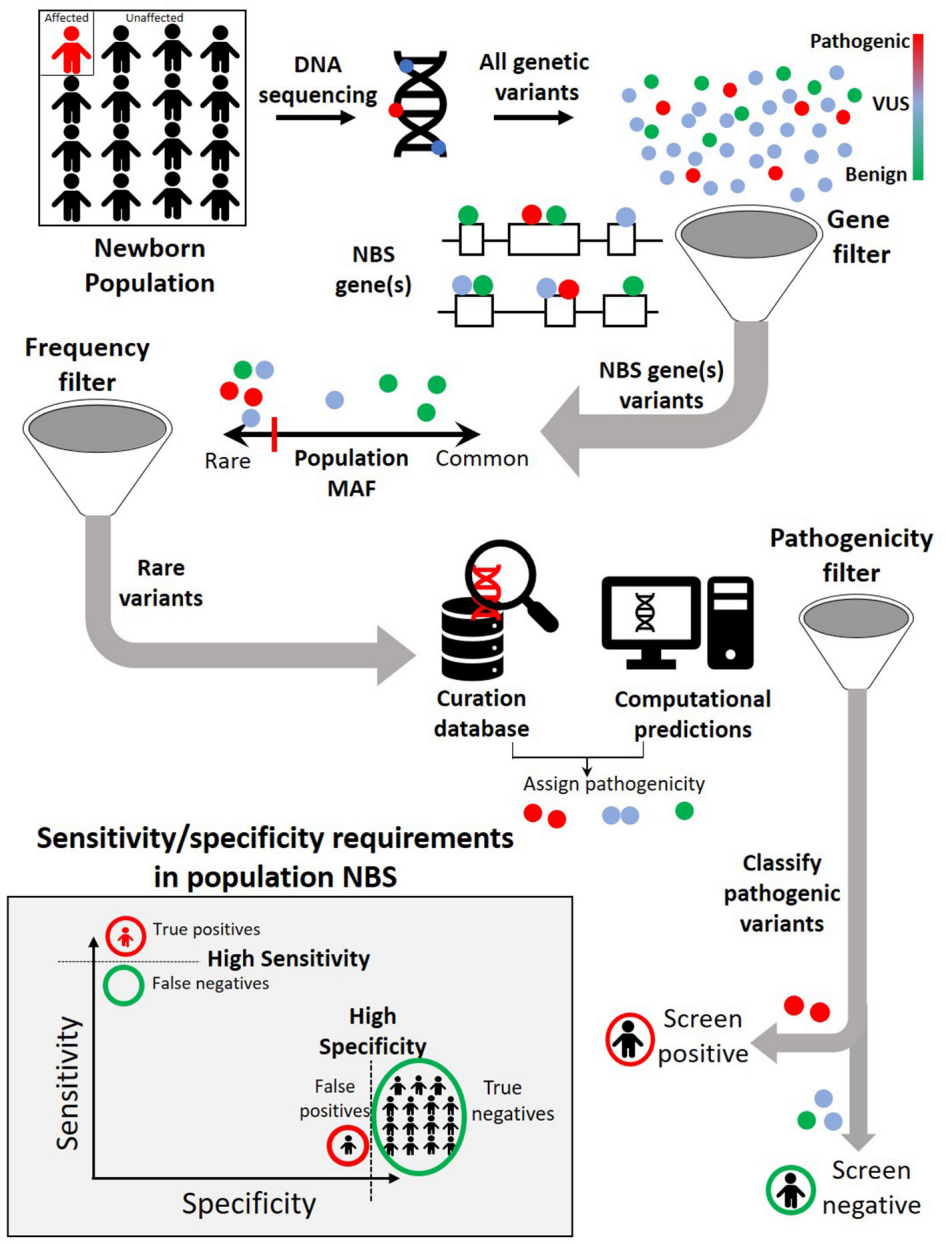
A typical genomic analysis pipeline in the context of newborn screening (NBS). Among all the variants observed in the newborn DNA sequence, only those occurring in previously identified NBS genes are considered further. Within NBS genes, rare variants are prioritized over common variants. A combination of curated pathogenic variant databases and computational prediction tools is utilized to assign variant pathogenicity and screen for individuals who carry such variants. For NBS, the pipeline will need to demonstrate both a high sensitivity (screen positive almost all newborns with disease) and a high specificity (screen negative almost all newborns without disease).

To effectively identify disease from DNA, the relevant disease-causing genetic variants should be both technologically detectable and clinically interpretable (Figure 2, Table 3). Historically, genetic tests have been limited in scope to a single or few genes associated with specific genetic conditions and performed using conventional technologies like Sanger sequencing of PCR-amplified coding regions of the gene(s) of interest. In recent years, high-throughput (next-generation) sequencing has greatly expanded the scope of genetic loci that can be technologically assayed in a single individual's genome. In particular, as noted in the previous section, WES is now a commonly used tool in diagnosis of various rare genetic disorders (61–66). However, challenges remain in applying WES or WGS to NBS, including the fact that capture and read coverage may be non-uniform and some disease-causing variants may be missed due to poor coverage. Recent simulations indicate that read mapping and variant calling in some NBS genes could be affected by homologous genomic regions (*CYP21A2, SMN1, CBS*, and *CORO1A*) (67). Whole genome sequencing expands the scope of detectable variants beyond coding regions. Besides revealing the ~98% of the non-coding genomic regions not visible to WES, WGS can provide a better view of the coding regions as well as more uniform coverage. Indeed, in some IEM, WGS revealed large deletions in IEM genes not observed by WES alone (55).

**Table 3:** Current primary technologies for detecting human genetic variants.

	**Scope**	**Advantages**	**Limitations**
Targeted gene panel	Captures variants within a few target genes (10s to 100s of genes)	- Disease-specific focus - High degree of customizability - Low cost and turnaround time	- List of all genes relevant to a disease needs to be explicitly defined beforehand - Needs to be updated as new disease genes are discovered
Whole exome sequencing	Captures variants within all exonic regions from the entire genome (~20,000 genes)	- Designed to capture all coding variants - Ideal for novel gene discovery in idiopathic conditions	- Misses non-coding and structural variants - Coverage and data quality can vary across genes
Whole genome sequencing	Captures variants from the entire genome	- More uniform coverage - Reveals non-coding and structural variants	- Lack of reliable tools to interpret non-coding variants - High cost, turnaround time and data storage requirements

Still, there are regions of the genome that are difficult to sequence using both conventional WES and WGS that rely on short-read sequencing (e.g., large insertions and deletions, tandem-repeat expansion, and complex chromosomal structural aberrations). Recent advances in long-read sequencing technologies could reveal such classes of variations to expand the repertoire for rare disease diagnosis. Even though such technologies are costly and data analysis methods are in early stages, there have been promising recent studies that have leveraged long-read sequencing to pathogenic variants in rare disease not detectable by conventional WES/WGS (68).

## Variant Interpretation and Current Challenges

Assuming the causative genetic variants are confidently detected, another major challenge for integrating genomics in NBS will be the clinical interpretation of genetic variants. To implicate pathogenic variants, the field leverages both curation of expert knowledge as well as prediction from computational tools.

Over the last few years, extensive clinical guidelines for variant interpretation have been developed, converging to ordinal five-tier “pathogenic” to “benign” labels for genetic variants (59). However, these guidelines currently do not accommodate the possibility that the same variant may need to be considered differently for specific diseases under different clinical contexts. In the context of NBS, similar to global collaborative efforts for harmonization of cutoff values in MS/MS data, collaborative efforts are needed to standardize and share variant classification and evidence across laboratories (69). In a recent study evaluating concordance of variant classification across nine laboratories (eight CLIA-accredited and one research laboratory), 54% of variants reached complete concordance, whereas 11% had a discordance that could affect clinical recommendation (70). After subsequent review of the discordant variants, the concordance increased to 84%. Even after review and data sharing, a significant proportion of existing variants remains of uncertain significance, labeled VUS, where additional evidence in the form of functional or computational studies are needed to resolve pathogenicity.

Several computational tools have been developed to interpret human genetic variants and predict their consequence on clinical phenotypes. Most of these tools aim to distinguish disease-causing pathogenic variants from those that are benign based on various existing information. Most tools predominantly leverage evolutionary conservation information, while others additionally incorporate physiochemical properties and protein structural data (71–74). Besides directly predicting clinical pathogenicity, specialized tools also exist for predicting various intermediate biochemical phenotypes including protein stability, RNA splicing, subcellular protein localization, protein interactions, and mechanism of action (75–79). Recently, meta-predictors have also emerged, which combine scores from several prediction tools using machine learning methods and report an integrated pathogenicity score (80, 81).

While tools can leverage the information about the knowledge of gene and protein function for coding variants, computational predictions of pathogenicity for noncoding variants can be even more challenging. Several tools have been developed to annotate non-coding regulatory regions distal (enhancers) or local (in promoters, untranslated regions or introns) to genes based on methods that integrate information from histone marks, transcription binding, gene expression, phylogenetic analyses, and chromatin accessibility (82–85). Other tools have emerged in this area that leverage machine learning techniques to integrate such annotations into pathogenicity scores (86–89). Besides single-nucleotide changes, several computational tools have also emerged to detect pathogenic structural variants from genomic data (90–92).

Despite over a hundred computational tools currently available for variant interpretation [recent catalog in ref (93)], challenges remain. Most computational tools train their algorithms in a supervised fashion across large databases of previously characterized human disease-associated and neutral variants. These tools typically use similar underlying training datasets, model features, and design principles, which leads to confounding issues of circularity and overfitting (94). Most tools assume that the properties that determine a variant's deleteriousness are generalizable across all genes. Yet, to make more accurate predictions of variant impact in a particular gene of interest, computational tools of the future may need to incorporate properties that capture the biological context specific to that gene. A recent work focused on computational tools specialized in predictions for a gene or gene family of interest has found that incorporating the context of an individual gene, biological pathway, and disease can improve quality of predictions (95, 96). The main challenge for gene-specific computational approaches, however, is the lack of sizable variant datasets for training on individual genes, particularly for rare diseases. A potential solution is emerging in the form of high-throughput functional assays that measure the variety of molecular and cellular consequences of all possible variants, in particular disease-relevant loci (97–99).

Another limitation of current variant interpretation tools is that they ignore the diplotypic context of an individual's genetic variant during both training and inference. The impact of a single pathogenic variant on the eventual clinical phenotypes is often modulated by other variants in the individual's genome, sometimes even within the same gene (100, 101). For example, in recessive disorders, the combination of pathogenic variants in both the paternal and maternal copies of a gene determines the clinical phenotypes and disease severity. To improve clinical utility of DNA analysis pipelines, the next generation of variant interpretation tools will need to incorporate the full diplotypic context in an individual's genome to predict both the likelihood as well as severity of diseases. For now, pathogenicity assertions about genetic variants using computational tools alone are not sufficient, and human review is still required for proper variant interpretation and return of genetic results.

## Sensitivity/Specificity Tradeoffs in Diagnosis Vs. Screening

Even though most genomic analysis pipelines for rare disease use similar sets of parameters, the optimal design and thresholds depend on the clinical context and application (102). Unlike diagnostic settings where DNA analysis is guided by phenotypic data to identify genetic variants that could explain an individual's clinical features, NBS is performed on asymptomatic newborns with no *a priori* phenotypes. The trade-offs of sensitivity and specificity can be different in these two contexts. Typically, diagnostic DNA pipelines report true positive rates (sensitivity) of around 25–60%, but the true negative rates (specificity) are often not reported. For population-scale NBS, both the sensitivity and specificity requirements are much stricter. Because most of the population will be unaffected in rare diseases typically screened for in the newborn period, even a 1% false positive rate for a screening test can translate to a large burden of false positive cases that require follow-up. Therefore, systematic exploration of parameters in analysis pipelines is necessary to achieve a balance between sensitivity and specificity that is best suited for screening (103).

As noted above, the NBSeq project, a retrospective study evaluating WES as a primary NBS test in an 8.5-year population-scale cohort for 48 IEM in California, achieved 88% overall sensitivity with a specificity of 98.4% (55). In comparison, current MS/MS analyte-based screening has a sensitivity and specificity of 99.0 and 99.8%, respectively, in the same cohort (69, 104–106). WES was therefore concluded to be insufficiently sensitive or specific as a general primary NBS test for IEM. The NC NEXUS study was performed in a smaller cohort and found similar results (54).

The appeal of genomic sequencing as a promising single test for all genetic diseases in the future should be reconciled with the possibility that the analytical performance of sequencing as a screening test may vary widely across different individual disorders. Such differences could arise from a range of factors, including limited prior genetic and clinical data, particularly in very rare conditions, as well as incomplete biological characterization of some genetic diseases. When grouped by prevalence, indeed the most common IEM (>2.5 per 10,000) had higher WES sensitivity of 91% and 78%, respectively, compared to the rarest (<0.04 per 10,000) (55).

An additional complexity arises from the fact that current databases of disease-associated genetic variants are largely Eurocentric. The larger proportion of previously uncharacterized variants in underrepresented populations could result in poorer performance of a sequencing-based screening test in such populations. Indeed, previous studies have demonstrated that variant misclassification in understudied populations can lead to genetic misdiagnoses with potential for exacerbating health disparities (107).

Besides analytical performance, bioinformatics analysis screening poses implementation challenges that may differ from a diagnostic scenario. Whereas, genomic analysis in diagnostic settings typically involves *ad hoc* rule-based pipelines with expert review of individual cases along with additional genomic data from other family members, NBS has to be performed at a population-level scale, requiring the analysis pipelines to be largely automated and streamlined.

## Ethical and Societal Considerations

Finally, even if all of the technical issues surrounding the application of next-generation sequencing to NBS are solved, social and ethical barriers to universal implementation will remain. Based largely on the ACMG criteria, many (perhaps most) genetic disorders would not qualify as candidates for NBS since early detection will not lead to specific therapy that will change the course of the disease and thus provide a broad societal benefit. This limitation, of course, does not address the potential non-specific benefits of early identification such as implementation of early intervention programs, appropriate prospective disease monitoring, and even family planning, as well as circumventing the prolonged diagnostic odyssey that individuals with rare diseases often face. The cost, not only of screening but also of follow-up, for affected individuals by government-funded programs must also be considered. In essence, WES/WGS screening would mandate a transfer of considerable health care resources from later-onset diagnostic expenses to neonatal screening and follow-up efforts. Additional ethical concerns also abound, most prominently the loss of the right to self-determination for the neonate identified with a later onset of disease.

## Conclusions

The evolution of NBS from testing one disease at a time to potentially identifying all possible diseases brings both great promise and challenges to modern health care systems. It is possible, perhaps even likely, that the overall cost to society will be reduced as NGS becomes more economical. However, the decision to adopt such a program must address a much more expansive set of issues that are likely to limit implementation in the near future, including identification of late-onset diseases for which no immediate therapy is available, improvement of turnaround time, and development of larger and multi-ethnic data sets of curated pathogenic variants that allow movement beyond variants of unknown significance. Societal concerns of genetic discrimination, especially as it relates to health insurance, must also be resolved. Rather, the use of NGS technology is more likely to continue to find its optimum use in diagnostic and follow-up settings, though moving to a primary role rather than as a tool of last resort as is now common. Instead, NGS panels and/or analysis focused on disorders with a significant neonatal, or even broader pediatric, footprint may play a role as a bridge to true WES/WGS NBS. Regardless, one of the key goals of traditional newborn screening, better care at lower cost, will ultimately be realized, translating next-generation sequencing to next-generation care.

## Author Contributions

AW, RG, JV, and AA contributed to conception and design of the article and wrote sections of the manuscript. AW compiled Table 1. RG compiled Table 2. JV designed Figure 1. AA designed Figure 2. All authors contributed to manuscript revision, read, and approved the submitted version.

## Conflict of Interest

AA is an employee of Illumina, Inc. The remaining authors declare that the research was conducted in the absence of any commercial or financial relationships that could be construed as a potential conflict of interest.
